# Ecological restoration for sustainable development in China

**DOI:** 10.1093/nsr/nwad033

**Published:** 2023-02-21

**Authors:** Bojie Fu, Yanxu Liu, Michael E Meadows

**Affiliations:** State Key Laboratory of Urban and Regional Ecology, Research Center for Eco-Environmental Sciences, Chinese Academy of Sciences, Beijing 100085, China; State Key Laboratory of Earth Surface Processes and Resource Ecology, Faculty of Geographical Science, Beijing Normal University, Beijing 100875, China; State Key Laboratory of Earth Surface Processes and Resource Ecology, Faculty of Geographical Science, Beijing Normal University, Beijing 100875, China; School of Geographic and Oceanographic Sciences, Nanjing University, Nanjing 210023, China; College of Geography and Environmental Sciences, Zhejiang Normal University, Jinhua 321004, China; Department of Environmental and Geographical Science, University of Cape Town, Rondebosch 7701, South Africa

**Keywords:** social-ecological system, landscape pattern, ecological process, ecosystem services, sustainable livelihoods, policymaking

## Abstract

Facing the need for transdisciplinary research to promote ecological restoration that achieves both social and ecological benefits, research on past restoration efforts that have directly or indirectly contributed to regional or national sustainable development warrants reassessment. Using China as an example, in this review, we address three basic research questions that can be summarized as follows: ecological restoration—of what, for whom and to what purpose? Accordingly, a ‘landscape pattern—ecosystem service—sustainable development’ co-evolutionary framework is proposed here to describe landscape-scale ecological restoration and its impact on landscape patterns and ecological processes, ecosystem services for human well-being, sustainable livelihoods and socioeconomic development. From the strategic pattern of national ecological security to the pattern of major projects to protect and restore major national ecosystems, the spatial pattern of China's ecological restoration is more geographically integrative. From major function-oriented zoning to systematic ecological protection and restoration, and for the purpose of achieving the Beautiful China Initiative, there are three stages of ecosystem services management: classification, synergy and integration, respectively. The difference in geographic processes should be considered in the key requirements of ecological restoration for China's five national strategies for regional sustainable-development strategies. Deepening understanding of the relationship between humans and nature in different geographical contexts is a scientific prerequisite to support policymaking related to ecological restoration. To promote greater harmony between humans and nature, we propose four important research directions: (i) understanding coupling processes among key components, (ii) identifying ecosystem service flows, (iii) evaluating social-ecological benefits and (iv) supporting adaptive management for regional sustainable development.

## Teaser

A ‘landscape pattern—ecosystem service—sustainable development’ co-evolutionary framework is built to review the landscape-scale ecological restoration and its impact on sustainable development in China.

## INTRODUCTION

Ecological restoration, defined as the process of assisting the recovery of an ecosystem that has been degraded, damaged or destroyed, is aimed at recovering ecosystem integrity that includes personal, cultural, socioeconomic and ecological values toward increasing social–ecological resilience [1]. The UN Decade on Ecosystem Restoration, from 2021 through 2030, delivers a rallying call for the protection and revival of ecosystems for the benefit of people and nature, and accordingly, it promotes the timely achievement of the sustainable development goals (SDGs) [2]. Considering the need for transdisciplinary research on ecological restoration that yields social and ecological benefits in the context of the UN Decade [3–5], a retrospective of past restoration efforts is needed at the national or regional scale to guide the future research agenda toward the goals of Agenda 2030 [6–8].

The contribution of ecological restoration to sustainable development relates mainly, although not exclusively, to SDG15 (Life on Land) [9,10]. Ideally, the SDGs will be achieved holistically, although it is clear that, while there are synergies between the goals, there are also tradeoffs [11,12], and these need to be considered in relation to ecological restoration. Moreover, given the marked differences between nations and regions, the following question arises: how can ecological restoration systematically promote sustainable development under different geographical contexts [13]? The social-ecological system concept, which lies at the core of sustainability science, emphasizes that people, communities, economies, societies and cultures are all embedded components of the biosphere across local and global scales [14]. To integrate ecological, economic and social processes in ecological restoration, the social-ecological systems approach has been widely employed, such as in (or in relation to) social-ecological restoration or social-ecological recovery [3]. Nevertheless, a synthetic conceptual framework in the social-ecological system context that incorporates co-evolution from the landscape pattern through to sustainable development and incorporating adaptive decision-making in ecological restoration is still lacking. With the ultimate goal of achieving sustainable development from a social-ecological perspective, three basic research objectives can be identified as: ecological restoration—of what, for whom and to what purpose?

China is addressing the issue of ecological restoration nationally, as it regards this as an important element of so-called ecological civilization [15]. While significant progress on land-system sustainability appears to have been achieved [16], reflecting on China's ecological restoration experience, and its social-ecological effects, may yield some important practical lessons for future adaptive restoration efforts toward the goal of sustainable development. However, although particular geographical areas may have been the object of such consideration, e.g. the Loess Plateau [17,18], there are few reviews of China's ecological restoration for sustainable-development actions at the national scale to date. Based on a social-ecological perspective, this review aims to summarize China's progress in ecological restoration for sustainable development and to propose a scientifically based ecological restoration agenda that promotes greater harmony between humans and nature in the country.

## RELATIONSHIP BETWEEN ECOLOGICAL RESTORATION AND SUSTAINABLE DEVELOPMENT

**Figure 1.**
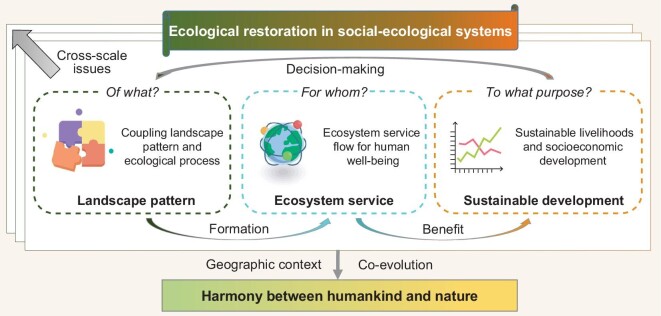
Conceptual relationship between ecological restoration and sustainable development.

Social-ecological systems have powerful reciprocal feedback and act as complex adaptive systems. The term ‘social-ecological’ emphasizes the integration of humans in nature and stresses that the delineation between society and the environment is artificial and arbitrary [19]. Three systematic, scientifically based steps are necessary to achieve sustainable development from ecological restoration: first, reciprocal effects between pattern and process need to be quantified; second, ecosystem services among various ecosystem functions should be identified, and the corresponding contribution of ecosystem services to human well-being must be acknowledged; and finally, adaptations and actions to regulate natural conditions should be harnessed for the promotion of sustainable development [20,21]. Although ecological restoration precisely represents this kind of regulating action, not all restoration approaches have the ability to promote sustainable development. The co-evolution of social-ecological systems can be considered to comprise the abovementioned three steps, corresponding to the questions of ecological restoration—of what, for whom and to what purpose (Fig. 1).

The first step in the co-evolution framework is ecological restoration on landscape pattern. The term ‘pattern’ generally refers to the spatial structure of landscape components, including their properties of size, type, number and distribution [22]. As opposed to merely describing the size of a restoration area or recording the number of trees [23], coupling landscape patterns and ecological processes yields more constructive, process-based guidance for ecological restoration measures at the landscape scale. For instance, the dynamics of soil erosion, carbon and hydrological cycle processes, and flow–sediment relationships in response to changes in landscape patterns, should be considered the key to monitoring the biophysical impacts of restoration efforts, such as the Grain-for-Green Program in China's Loess Plateau [18].

The second step in the co-evolution framework focuses on ecological restoration for ecosystem services and also for human well-being. Ecological processes are too often ignored or mismanaged, but landscape components can be structured to deliver ecosystem services that maintain key ecological processes while simultaneously meeting human needs and well-being [24]. For instance, significant conversions of farmland to woodland and grassland resulted in enhanced soil conservation and carbon sequestration in China's Loess Plateau [25]. The research challenge lies in understanding the spatially different and often cross-scale relationships between ecosystem services and human well-being following ecological restoration, since there are teleconnections between them, while the flow from the ecosystem service supply to fulfilling human needs is often unclear [26].

The final step in the co-evolution framework is ecological restoration to sustainable development with cyclical decision-making for adaptive management, which moves toward the ongoing dual outcomes of sustainable livelihoods and socioeconomic development. Social and economic issues, such as limited access to markets and input resources, weak governance and lack of knowledge around alternative production technologies, frequently constrain the options available to communities in degraded landscapes [27], such that ecological restoration may encourage local investment and lead to employment opportunities under adaptive management combined with socioeconomic development [28]. In addition, the costs of improving ecosystem service delivery in an ecological restoration project may be considered, at least partially, as leverage for local sustainable livelihoods [29,30]. It should be noted that the contribution of ecological restoration to markets and livelihoods is not unidirectional, as the issues in sustainable livelihoods and socioeconomic development also drive decision-making in ecological restoration. Therefore, if the vision of ‘harmony between humankind and nature’ is to be realized, co-evolutionary pathways should be highlighted, including both the flow of ecosystem services from nature to humans and decision-making around ecosystem services delivery from humans to nature.

## PROGRESS OF ECOLOGICAL RESTORATION IN CHINA

### Ecological restoration effects on landscape pattern

Since the Three-North Shelterbelt Development Program began in 1978, China has implemented more than a dozen interprovincial ecological restoration programs (Box 1). Most of the main landscape components of China's major ecological restoration projects were based on terrestrial ecosystem types, including forest, grassland and cropland [16]. In addition to those components, the wetland, marine and coastal landscapes have recently received attention, and large-scale ecological restoration projects have been established. During the selection of these programs, we found that the terms ‘reclamation’ and ‘restoration’ were not explicitly separated in some of the plans at the time of their implementation, e.g. the National Land Consolidation Program; note that the mining land reclamation programs are not included in this review.

In China, the integrality of landscape patterns was described as ‘a community of life including mountains, rivers, forests, farmlands, lakes, grasslands and deserts’. From 2016 to 2020, 26 pilot projects, collectively named the Ecological Protection and Restoration of Mountains, Rivers, Forests, Farmlands, Lakes and Grasslands Program, were implemented, aiming at integrative ecological restoration of landscapes. With the success of the pilot projects, 19 projects, collectively named the Integrative Ecological Protection and Restoration of Mountains, Rivers, Forests, Farmlands, Lakes, Grasslands and Deserts Program, were implemented in 2021 and 2022.

The achievements of China's ecological restoration projects are apparent. Based on the Chinese government's white paper of Forest and Grassland Resources and Ecological Status in China 2021 [31], China has 231 million hectares of forest, with a forest coverage rate of 24.02%. The grassland area is 264.5301 million hectares, the comprehensive vegetation coverage of grassland is 50.32%, and the total output of fresh grass is 595 million tons. The total carbon storage of forest and grass is 11.443 billion tons. In addition, China has 56.2938 million hectares of wetlands.

**Box 1.** China's major ecological restoration programs.

 

1. **The Three-North Shelterbelt Development Program**
2. **Aims:** Halt desertification and improve the environment.
3. **Planned investment:** 57.68 billion yuan (1st Phase to 4th Phase).
4. **Start time:** 1978
5. **National Key Construction Program for Soil and Water**
6. **Aims:** Control soil erosion, improve agricultural production conditions, ecology and the environment.
7. **Planned investment:** 14.54 billion yuan (1st Phase to 5th Phase).
8. **Start time:** 1983
9. **The Shelterbelt Development Program in Five Regions** (Yangtze River Shelterbelt, Coastal Shelterbelt, Pearl River Shelterbelt, Taihang Mountain Greening and the Plain Greening)
10. **Aims:** Arrest the deterioration of the ecology and environment of the Yangtze River, Pearl River and their coastal areas.
11. **Planned investment:** 258.42 billion yuan (1st Phase to 3rd Phase).
12. **Start time:** 1987
13. **Comprehensive Agricultural Development Program**
14. **Aims:** Raise the quality of life in the countryside, and expedite land reform and long-term food security.
15. **Planned investment:** 32.65 billion yuan (1st Phase).
16. **Start time:** 1988
17. **National Land Consolidation Program**
18. **Aims:** Manage the area of cultivated land, improve its utilization and increase land revenues.
19. **Planned investment:** 2.633 trillion yuan (1st Phase and 2nd Phase).
20. **Start time:** 1997
21. **Natural Forest Conservation Program**
22. **Aims:** Protect and restore natural forests.
23. **Planned investment:** 320.22 billion yuan (1st Phase and 2nd Phase).
24. **Start time:** 1998
25. **Grain for Green Program**
26. **Aims:** Increase forest cover, alleviate soil erosion, conserve biodiversity and increase rural household income.
27. **Planned investment:** 212.81 billion yuan (1st round).
28. **Start time:** 1999
29. **Program of the Base Construction of Fast-Growing and High-Yielding Timber Forest**
30. **Aims:** Remedy the decline in timber supply.
31. **Planned investment:** 71.8 billion yuan (1st Phase to 3rd Phase).
32. **Start time:** 2001
33. **Central Government Forest Ecosystem Compensation Fund Program**
34. **Aims:** Protect species, improve the living environment and maintain ecological balance.
35. **Planned investment:** 80.1 billion yuan (2001 to 2014).
36. **Start time:** 2001
37. **The Sandification Control Program for Areas in the Vicinity of Beijing and Tianjin**
38. **Aims:** Improve and optimize the ecological environment and reduce the risk of sandstorms.
39. **Planned investment:** 143.66 billion yuan (1st Round and 2nd Round).
40. **Start time:** 2001
41. **Ecological Protection and Construction on Qinghai-Tibet Plateau** (Ecological Protection and Construction at the Three River Source Region in Qinghai, and Protection and Construction of the Ecological Security Barrier in Tibet)
42. **Aims:** Reduce desertified land and degraded grassland, and increase forest coverage.
43. **Planned investment:** 143.66 billion yuan (1st Round and 2nd Round).
44. **Start time:** 2005
45. **National Wetland Protection Program**
46. **Aims:** Maintain the ecological characteristics and basic functions of the wetland ecosystem.
47. **Planned investment:** 90.04 billion yuan (2006 to 2010).
48. **Start time:** 2006
49. **Rocky Desertification Comprehensive Treatment Program in Karst Areas**
50. **Aims:** Curb the expansion of desertification in rocky environments, improve the ecological environment and maintain national ecological security, promote national unity and social harmony.
51. **Planned investment:** 11.9 billion yuan (1st Phase).
52. **Start time:** 2008
53. **The Grassland Ecological Protection Subsidies and Awards Program**
54. **Aims:** Protect national ecological security, promote the development of pastoral areas and herders’ incomes, maintain national unity and stability in the border area, coordinate the development of urban and rural areas.
55. **Planned investment:** ∼77 billion yuan (1st Round).
56. **Start time:** 2011
57. **Cultivated Land Quality Protection and Promotion Program**
58. **Aims:** Enhance national food security, and the quality, safety and ecological sustainability of agricultural production.
59. **Start time:** 2015
60. **Marine Ecological Protection and Restoration Program** (Blue Bay Initiative)
61. **Aims:** Improve the ecological environment function of coast, sea area and island.
62. **Planned investment:** 3 or 4 billion yuan per city.
63. **Start time:** 2016
64. **Integrative Ecological Protection and Restoration of Mountains, Rivers, Forests, Farmlands, Lakes, Grasslands and Deserts Program**
65. **Aims:** Enhance the overall self-recovery capacity, stability and quality of natural ecosystems, as well as the overall enhancement of the supply capacity of ecological products.
66. **Planned investment:** ∼5 billion yuan per project.
67. **Start time:** 2016

The studies also clearly demonstrate changes in landscape pattern and related ecological processes emanating from China's large-scale ecological restoration projects. Satellite data at the national scale (2000–2017) show that vegetation greenness in China has greatly increased in the last two decades, and the country alone accounts for 25% of the global net increase in leaf area, of which 42% is from forests [32]. Wang *et al.* used Landsat images to demonstrate a substantial increase in salt marsh areas since 2012 in China's coastal wetlands, driven by reduced anthropogenic activities and increased conservation and restoration efforts [33]. It has been reported that >45% of China's drylands experienced statistically significant land improvement or vegetation greenness from the 1980s to 2015, attributable to, among other interventions, afforestation and desert regeneration efforts in 13 ecological conservation and restoration programs [34]. Based on sediment load observations, Wang *et al.* demonstrated that large-scale vegetation restoration in the Loess Plateau substantially reduced soil erosion from the 1990s onward [35].

Toward longer-term conservation of entire landscapes, the establishment of ecological red lines can safeguard China's vast biodiversity, environmental resources and ecosystem services [36]. Protection of areas by ecological red lines is part of the newly revised Environmental Protection Law of China and is listed as one of the priority actions to achieve ecological civilization [37]. The ecological red lines can be defined as the minimum space that needs the strictest protection to improve ecological functions, to ensure the sustainable supply of ecological goods and services [38,39]. By 2022, >25% of China's territory was covered by areas of ecological red lines, which are believed to powerfully relieve or reverse ecosystem degradation in ecologically important and sensitive landscapes.

**Figure 2.**
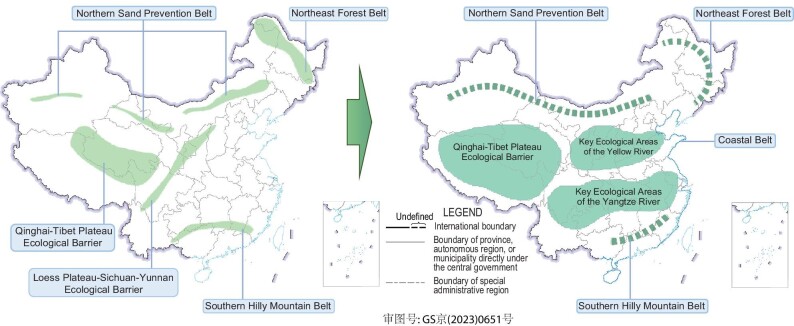
The strategic pattern of national ecological security and the pattern of major projects to protect and restore major national ecosystems.

Moreover, from the strategic pattern of national ecological security to the pattern of major projects to protect and restore major national ecosystems, the pattern of China's ecological restoration is becoming more geographically integrated (Fig. 2). China's Master Plan for Major National Projects to Protect and Restore Important Ecosystems (2021–2035) has demonstrated a national pattern of ‘three key areas and four belts’. This pattern considered the integrity of geographical units more than the past strategic pattern of national ecological security, abbreviated as ‘two barriers and three belts’. Based on hierarchical ecological restoration planning across nation, province, prefecture-level city and county, China's landscape pattern will continue to be optimized by ecological restoration until 2035, mainly in the Qinghai-Tibet Plateau Ecological Barrier, Key Ecological Areas of the Yellow River, Key Ecological Areas of the Yangtze River, Northern Sand Prevention Belt, Northeast Forest Belt and Coastal Belt.

Although the main aims of these programs were to improve the environment and enhance human livelihoods, the approach adopted was not always compatible with comprehensively achieving sustainable development. Indeed, the social-ecological systems approach was not typically employed in the planning and implementation of most of these programs, so that they cannot be regarded as the final word on China's contribution to ecological restoration, which remains a work in progress. In the following sections, we consider both the contributions and limitations of these programs on the basis of the scientific literature.

### Ecological restoration for ecosystem services

The ecological restoration programs in China have greatly influenced ecosystem services, especially carbon sequestration, soil retention and water yield, and their interactions, and it is clear that some spatiotemporal trade-off relationships need to be considered.

Ecological restoration has brought about extensive increases in carbon sequestration in China. Lu *et al.* estimated that a carbon drawdown of 74 Tg C y^−1^ resulted from the implementation of China's six national key ecological restoration programs between 2000 and 2010 [40]; while in a more recent study, mitigation arising from natural climate solutions was estimated at 0.6 (0.5–0.7) PgCO_2_e yr^−1^ between 2000 and 2020 [41]. As an example, Zhang *et al.* reported that depopulation in rural China (−14 million people yr^−1^ between 2002 and 2019) was associated with the development of a substantial aboveground carbon sink of 0.28 ± 0.05 PgC yr^−1^ [42]. Liu *et al.* estimated that the 1st Phase of the Natural Forest Conservation Program provided 12.71 Tg C·yr^−1^ net carbon sequestration; and the Grain for Green Program provided 18.50 Tg C·yr^−1^ net carbon sequestration [43].

However, increased water consumption related to ecological restoration programs has been a cause for concern, especially in drylands. Cao *et al.* estimated that at the national scale, China's afforestation may increase water consumption by 559–2354 m^3^/ha annually compared with natural vegetation without restoration [44]. Zhao *et al.* observed the significant depletion of terrestrial water storage following ecological restoration in the Mu Us Desert [45]. Based on estimates of evapotranspiration and human water demand, Feng *et al.* estimated that net primary productivity was close to growth limits (∼400 g C m^−2^ yr^−1^) considering the water resource capacity in revegetated areas of the Loess Plateau [46]. Considering the temporal aspects of trade-offs, Li *et al.* conclude that while soil erosion has been reduced by afforestation in the Haihe River Basin, surface runoff has declined significantly after a time lag of 18 years, substantially limiting the overall desired benefit [47].

**Figure 3.**
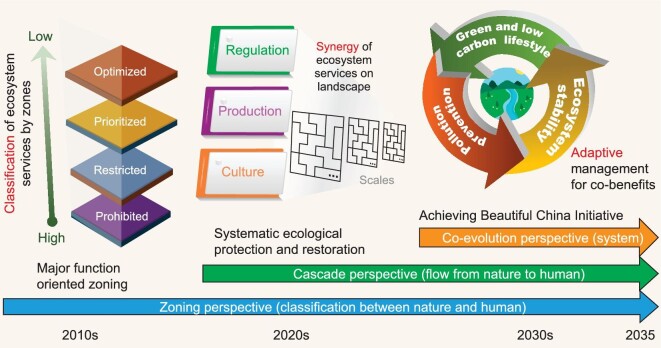
The three stages of ecosystem services management from major function-oriented zoning to systematic ecological protection and restoration and onwards to the achievement of the Beautiful China Initiative.

China's ecological restoration has improved multiple ecosystem services and therefore has the ability to benefit human well-being. With regard to the spatiotemporal trade-off relationships between ecosystem services, geographical differences should be considered, and the coupling mechanism between humans and nature in a geographic context should be scientifically revealed to holistically benefit human well-being. From major function-oriented zoning to systematic ecological protection and restoration toward achievement of the Beautiful China Initiative, we consider three stages of ecosystem services management: classification, synergy and integration (Fig. 3).

The first stage was China's major function-oriented zoning in 2010, which was planned to classify regional major functions for human well-being in a geographic context [48]. This plan can provide a classification of ecosystem services for geographical zones. The optimized-oriented zone is an urbanized area that requires optimization of the supply–demand relationship of ecosystem services to maintain human well-being. The prioritized-oriented zone is an urbanization area with high potential for development under the appropriate harnessing of ecosystem services. The restricted-oriented zone is the source area of provision services and regulation services. The prohibited-oriented zone is a vulnerable area where resource exploitation damages local ecosystem services. However, this stage took only a zoning perspective with regard to the classification of nature and humans, while trade-off relationships and flows were not adequately considered in this classification.

The second stage is the implementation of China's Master Plan for Major National Projects to Protect and Restore Important Ecosystems (2021–2035), which promotes systematic ecological protection and restoration at the landscape scale. Guided by the ecological civilization concept of ‘a community of life including mountains, rivers, forests, farmlands, lakes, grasslands and deserts’, recent ecological restoration projects have the potential to enhance the synergy of typical ecosystem services in landscapes at different scales. During this stage, a cascade perspective on the flow from nature to humans is constructed. Synergy is advocated, since the flow from ecosystem service supply to the fulfillment of human needs is still unclear in many of China's ecological restoration projects, and how these synergetic benefits then provide enduring incentives (or not) for good management has not been well understood.

The third stage is achieving the Beautiful China Initiative in 2035, when the integration of ecosystem service supply, demand and flows for co-benefits can be realized. The integration of ecosystem services is not an elimination of trade-off relationships but an integrative enhancement of all required ecosystem services for co-benefits involving human well-being in a geographical context. Towards this stage, a co-evolutionary perspective on social-ecological systems is required for understanding how the benefits of ecosystem service flows are translated into social, economic and policy incentives, so that locals may benefit from the restoration actions and thus act to support adaptive management, rather than passively accepting the policy. A new set of ecological restoration practices that explicitly considers human–nature dynamics to ensure these incentives are maintained in perpetuity should emerge at this stage.

### Ecological restoration for the purpose of sustainable development

Research suggests that there existed local win−win synergies between ecosystem health and sustainable livelihoods and/or socioeconomic development. However, the identification of such win−win solutions for regional policymaking is still in progress, as benefits and incentives change across scales.

There is clear evidence that at least some ecological projects have successfully benefited local livelihoods. In critically evaluating the Paddy Land to Dry Land Program in Beijing, Zheng *et al.* report that both regulating services and household income benefited from implementation of a system whereby water users pay upstream landholders [49]. Similarly, Zheng *et al.* simulated alternative land-use scenarios to identify win–win outcomes for regulating services and rubber production in the Ecosystem Function Conservation Area of Hainan Island [50], and Hou *et al.* concluded that China's Grassland Ecological Compensation Policy improved both grassland quality and had a large positive effect on herder incomes [51].

However, ecological restoration programs are not always universally beneficial. Li *et al.* outline how significant short-term costs for poorer households prevented residents from participating because they lacked the resources to afford relocation in the Relocation and Settlement Program in the southern Shaanxi Province [52]. In contrast, resettled households transformed livelihood activities from traditional agriculture and forestry labor to off-farm activities that yielded increased income after relocation, which is a win−win situation [53]. Cao *et al.* proposed an income threshold associated with the poverty trap, whereby sustainable livelihoods may be uplifted to achieve a win–win solution if their incomes are raised above a particular minimum amount [28].

There is also some local evidence that synergies exist between ecosystem health and socioeconomic development. Following the implementation of a water diversion project in the Heihe River Basin, the deterioration of ecosystems downstream was substantially alleviated, sustaining both ecological health and socioeconomic development [54]. Cao *et al.* estimated that the returns on investment from the Three-North Shelter Forest System Project, the Natural Forest Conservation Program and the Grain-for-Green Program were 29.3%, 328.9% and 77.0%, respectively [55]. However, quantitative cost−benefit analyses of China's numerous ecological restoration programs are largely lacking.

**Figure 4.**
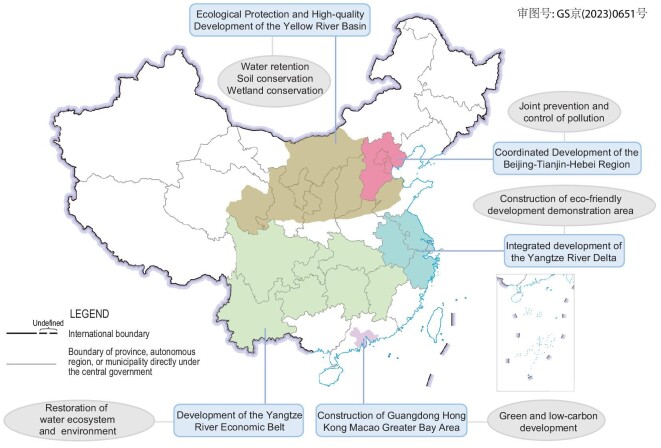
The key requirements for ecological restoration for the five national strategies for regional sustainable-development strategies.

Taking the five national strategies for regional sustainable-development strategies as examples, geographical differences in the key requirements for ecological restoration for regional sustainable development are highlighted (Fig. 4). For the strategy of Ecological Protection and High-Quality Development of the Yellow River Basin, upstream water retention, midstream soil conservation and downstream wetland conservation were highlighted. For the strategy of the Development of the Yangtze River Economic Belt, the restoration of the water ecosystem and environment is more extensive, e.g. a fishing ban for a decade (2021–2030). Joint prevention and control of pollution is the primary environmental requirement in the strategy of Coordinated Development of the Beijing-Tianjin-Hebei Region. The construction of an eco-friendly development demonstration area is an objective of the strategy of integrated development of the Yangtze River Delta, and green and low-carbon development is an objective of the strategy of construction of the Guangdong Hong Kong Macao Greater Bay Area. Although these three regional requirements cannot be solved solely by ecological restoration, enhanced ecosystem services, such as carbon sequestration, water quality purification and heat regulation, can contribute to regional sustainable development. Given that benefit flows, and their recipients, change across scales in ecological restoration [44–47], research should focus on how to transfer benefits from those who gain to those who may lose, especially in the context of regional sustainable-development strategies.

## FUTURE RESEARCH NEEDS

**Figure 5.**
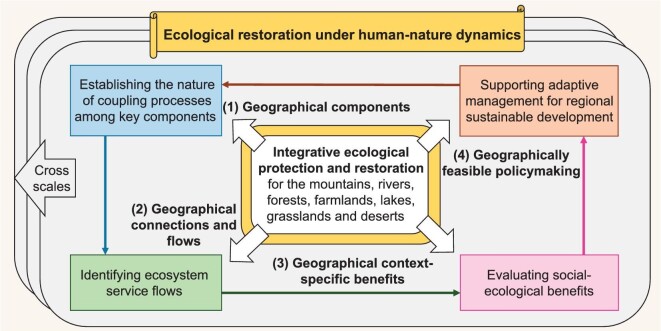
Research needs for ecological restoration in a geographic context.

Considering China's vast terrestrial area, diversity of ecosystem types and large differences in the levels of rural development, an in-depth scientific understanding of the human–nature relationship in different geographical contexts is a prerequisite for supporting policymaking on ecological restoration for sustainable development. Accordingly, four geographical research perspectives for integrative ecological protection and restoration are highlighted here, including establishing the nature of coupling processes among key components, identifying ecosystem service flows, evaluating social-ecological benefits and supporting adaptive management for regional sustainable development (Fig. 5).

### Coupling processes among key components

Selection of the most relevant components in establishing coupling processes is essential for minimizing possible measurement errors in a social-ecological system. However, selecting the correct components engaged in ecological and social processes in a particular geographical context and understanding the coupling relationships among these processes is generally impossible at the start, since social-ecological systems are complex systems. In working towards the goal of ecological restoration, setting clear hypotheses, establishing a monitoring and learning regime to test and track these hypotheses over time, and using these to drive (and constrain) an adaptive management (and policy) approach are needed.

Based on different geographical components, three frontiers of coupling can be identified in the above-mentioned approach. The first is how to decide, initially, what the key components engaged in coupled processes are. For example, what components form part of the ecological health index—and how/why were they selected? The second is in the monitoring and learning regime; how would key feedback from ecological restoration relating to, for example, biogeochemical cycles or social behavior be identified? Relationships between restorative actions on ecosystem attributes, such as water resources or carbon sequestration, generally require further research [56]. The third frontier lies in understanding how ecological and social processes influence landscape patterns after ecological restoration for adaptive management; for example, disturbances, such as warming, irrigation and grazing, further affect the restored landscape and thus impact ecosystem stability.

### Identification of ecosystem service flows

Research on the response of ecosystem services to ecological restoration in China has focused mainly on how they have been influenced by landscape change. However, human well-being is still inadequately explored. The concept of ecosystem service flow is not in itself new [57], although spatially explicit flows have rarely been included in ecological restoration research. Therefore, to uncover the real contribution of restoration projects to human well-being, establishing the details of ecosystem service flows is an essential research objective.

Considering geographical connections and flows, we propose two frontiers. First, it is imperative to identify ecosystem service flows from a social-ecological network perspective. This involves measuring dispersal-related flows, such as animal pollination and migration, or material flows, such as the extraction and transport of sand for building materials [58], and it must also include measurement of the potential flow of ecosystem services to households [59]. The second frontier is detecting potential ecosystem service trade-offs at different spatial scales, from local to national, and across short and long temporal scales, based on ecosystem service flows. Depending on the spatial scale, ecosystem service supply and demand can be linked to establish internal matching of proximal benefits and cross-regional matching of longer-range benefits, which can then be used to determine the costs of ecosystem services in an ecological restoration project.

### Evaluation of social-ecological benefits

Social-ecological benefits need to be evaluated in a holistic, geographical context. If, as may be the case, restoration ecologists and social scientists remain strictly within their own research field, this may prevent the kind of comprehensive assessment of restoration efforts on ecosystem health and socioeconomic sustainability that a social-ecological perspective requires [60]. Building a systematic model including all the essential components in a social-ecological system is advocated so that any change in a particular factor reveals the associated responses of both ecological and social benefits in the restoration program.

Over and above the usual challenges of data acquisition and complexity of coding in model development, there are two frontiers in appraising any geographical context-specific benefits. First, there is the context-specific parameter rule, including both community-level parameters within household information and landscape-level parameters within geographical features [61]. The second frontier involves elucidating the primary and secondary relationships of benefits. Focusing on the enhancement of prior ecosystem services to meet global and local demands of current and future social-ecological benefits, ecologists and social scientists participating in model development should understand the primary relationships of benefits and gradually improve the standard analytical process for multi-objective ecological restoration [62], which should not ignore sufficient communication with policymakers and practitioners.

### Adaptive management for regional sustainable development

Policies aimed at improving environmental conditions that simultaneously advance local sustainable livelihoods must be supported by both accurate assessment of the implemented restoration projects and comprehensive predictions of how the designed restoration projects will unfold. Although remote sensing data sets provide abundant, easily accessible and detailed information on essential components related to ecosystem health, many assessments of the effect of ecological restoration policy on local sustainable livelihoods and socioeconomic development have not been adequately performed. In addition, notwithstanding that robust science must play a central role in policymaking for ecological restoration, a purely technical approach cannot be successful. Ecologists and social scientists as well as policymakers and practitioners, need to work together to resolve the challenges.

Accordingly, two research frontiers can be highlighted to promote ecologically sensitive and geographically feasible policymaking that fosters regional sustainable development. First, high-quality, reliable and science-based assessments of the effectiveness of ecological restoration policies are required [63]. The key need is for robust, scientific assessments of the impacts of restoration projects on the environment and on society under different geographic contexts. Second, adaptive planning strategies should be based on lessons learned from previous sustainable-development outcomes. In addition to collaboration among ecologists and social scientists, good communication is needed with policymakers, managers and practitioners for effective ecosystem management that promotes sustainability in the broader context. Geographically feasible policymaking should meet the needs of practitioners and policymakers in related fields, such as natural resource management, environmental protection, animal husbandry and rural development.
